# Synthesis and Characterization of Zinc/Iron Composite Oxide Heterojunction Porous Anode Materials for High-Performance Lithium-Ion Batteries

**DOI:** 10.3390/molecules28227665

**Published:** 2023-11-19

**Authors:** Ruixiang Wang, Yanyang Wang, Wei Xiong, Jiaming Liu, Hui Li

**Affiliations:** 1 Ganzhou Engineering Technology Research Center of Green Metallurgy and Process Intensification, Department of Materials, Metallurgy and Chemistry, Jiangxi University of Science and Technology, Ganzhou 341000, China; 9120030519@jxust.edu.cn (R.W.); 6120190228@mail.jxust.edu.cn (Y.W.); 1320211693@mail.jxust.edu.cn (W.X.); 2 Farasis Energy (GanZhou) Co., Ltd., Ganzhou 341000, China

**Keywords:** ZnO/ZnFe_2_O_4_/NC, heterojunction, lithium-ion batteries, anode materials, long-term cycling stability

## Abstract

Environmental pollution caused by the use of fossil fuels is becoming increasingly serious, necessitating the adoption of clean energy solutions. Lithium-ion batteries (LIBs) have attracted great attention due to their high energy density and currently occupy a dominant commercial position. Metal oxide materials have emerged as promising anode materials for the next generation of LIBs, thanks to their high theoretical capacity. However, the practical application of these materials is hindered by their substantial volume expansion during lithium storage and poor electrical conductivity. In this work, a zinc/iron bimetallic hybrid oxide composite, ZnO/ZnFe_2_O_4_/NC, is prepared using ZIF-8 as a precursor (ZIF-8, one of the metal organic frameworks). The N-doped porous carbon composite improves the volume change and optimizes the lithium-ion and electron transport. Meanwhile, the ZnFe_2_O_4_ and ZnO synergistically enhance the electrochemical activity of the anode through the built-in heterojunction to promote the reaction kinetics at the interface. As a result, the material delivers an excellent cycling performance of 604.7 mAh g^−1^ even after 300 cycles of 1000 mA g^−1^. This study may provide a rational design for the heterostructure and doping engineering of anodes for high-performance lithium-ion batteries.

## 1. Introduction

To this day, the extensive use of fossil fuels in thermal power generation has caused serious environmental pollution. Searching for new clean energy sources to replace traditional fossil fuels has become an urgent issue [1,2,3]. Among various electrochemical power sources such as lithium-ion batteries (LIBs), lead–acid batteries, nickel–metal hydride batteries and nickel–cadmium batteries, which are commonly used by people, lithium-ion batteries have become a new answer to solve the energy problem and the environmental pollution problem because of their high energy density, strong power stability, low environmental pollution and lack of memory benefits [4,5,6,7]. However, the commercialized graphite anode has a low theoretical capacity, which makes it difficult to meet the energy density requirements of the new generation of LIBs. Therefore, conversion-type reaction anode materials, such as transition metal oxides (TMOs) with higher theoretical capacity, have become a promising research direction. Nevertheless, the instability and poor conductivity of TMO anodes due to volume expansion during lithium-ion insertion/extraction can seriously hinder their electrochemical performance [8,9,10,11]. Based on this issue, the rational design of micro- and nanostructures has been proposed as an effective strategy [12]. Heterostructured composites, such as active materials coupled with porous carbon materials, have shown potential in solving the above problems [13,14,15]. The composite of porous carbon-based materials not only enhance lithium-ion and electron transport, but also mitigate the volume change in the active material, maintaining the integrity of the anode during the discharge/charge process [16,17,18].

Metal–organic framework materials (MOFs), as porous crystalline materials formed by metal ions and organic ligands, have been extensively studied as precursors to prepare nanostructured metal oxides and metal oxide/carbon composites for lithium-ion battery anodes [19,20,21,22]. The calcination of MOFs in air can produce metal oxides retaining the original framework morphology, whereas calcination in an inert atmosphere leads to metal oxide/carbon composites, in which the carbon coating helps accommodate volume changes and enhances conductivity [23,24]. For instance, Wu et al. successfully synthesized perfect octahedral morphology porous CuO hollow-structured material by the simple annealing of copper-based metal–organic framework (MOF) templates, delivering excellent cycling stability [25]. Han et al. successfully prepared a porous nitrogen-doped carbon-coated Co_3_O_4_ fish scale structure material by the chemical conversion of nitrogen-rich Co-MOF at 500 °C in a nitrogen atmosphere. This sample can maintain a stable value of specific capacity around 612 mAh g^−1^ after 500 cycles at a current of 1000 mA g^−1^ due to the interaction between the porous Co_3_O_4_ nanoparticles and the N-doped carbon coating, and it shows excellent electrochemical performance as an anode material for lithium-ion batteries [26]. Compared to monometallic oxides, bimetallic transition metal oxides are currently the focus of researchers’ attention due to their unique synergistic effect advantages [27,28,29]. As examples, Chu et al. synthesized a hollow NiCo_2_O_4_ nanowire based on Ni/Co-MOFs by direct pyrolysis in air, which showed a capacity of 1310 mAh g^−1^ after 100 cycles at 100 mA g^−1^ [30]. Mei et al. synthesized hollow octahedral ZnFe_2_O_4_@C nanocomposites using Zn/Fe-MOFs as precursors, demonstrating an ultra-long cycling stability of 918 mAh g^−1^ after 800 cycles at a current density of 3 A g^−1^ [31]. ZnO/ZnFe_2_O_4_@reduced graphene oxide (RGO) nanocomposites have been successfully synthesized through annealing treatment of Zn/Fe MOF-5@GO composites [17]. They are used as anodes for lithium-ion batteries with excellent performance. In addition, synthesis can also be based on Prussian Blue Analogue (PBA). For example, Xing et al. directly prepared high-performance anode materials with ZnO/ZnFe_2_O_4_ core–shell structures using PBA derivatives [32]; Yuan et al. synthesized high-performance ZnO/ZnFe_2_O_4_@C by adding dopamine on the basis of oxides formed by PBA materials and anode material [33]. Marcella Bini et al. reviewed ZnFe_2_O_4_ as a high-performance anode material for lithium-ion batteries [34]. All these works provide ideas for the development of anode materials with effective nanostructures for LIBs, which can help in the design of various metal oxide/carbon composites of interest for energy storage applications.

In this work, a bimetallic oxide heterostructure carbon–nitrogen composite, ZnO/ZnFe_2_O_4_/NC, was obtained by the two-step heat treatment of ZIF-8. The combination of ZnFe_2_O_4_ and ZnO nanoparticles was designed to synergistically enhance the electrochemical activity of the anode through the in-built heterojunction between the two phases, promoting reaction kinetics at their interface. Meanwhile, the porous carbon–nitrogen groups derived from ZIF-8 as a matrix to mitigate the volume change while also enhancing the ion/electron transport. Under the synergistic effect of the unique hybrid nanostructures and compositional features, the ZnO/ZnFe_2_O_4_/NC composites exhibited excellent cycling performance, with capacities of 812.8 mAh g^−1^ after 100 cycles of 200 mA g^−1^ and 604.7 mAh g^−1^ after 300 cycles of 1000 mA g^−1^. This work demonstrates the promise of rationally heterostructured anodes with bimetallic oxides and embedded carbon matrices for high-performance lithium-ion batteries.

## 2. Results and Analysis

### 2.1. Chemical and Structural Characterization

**Figure 1:**
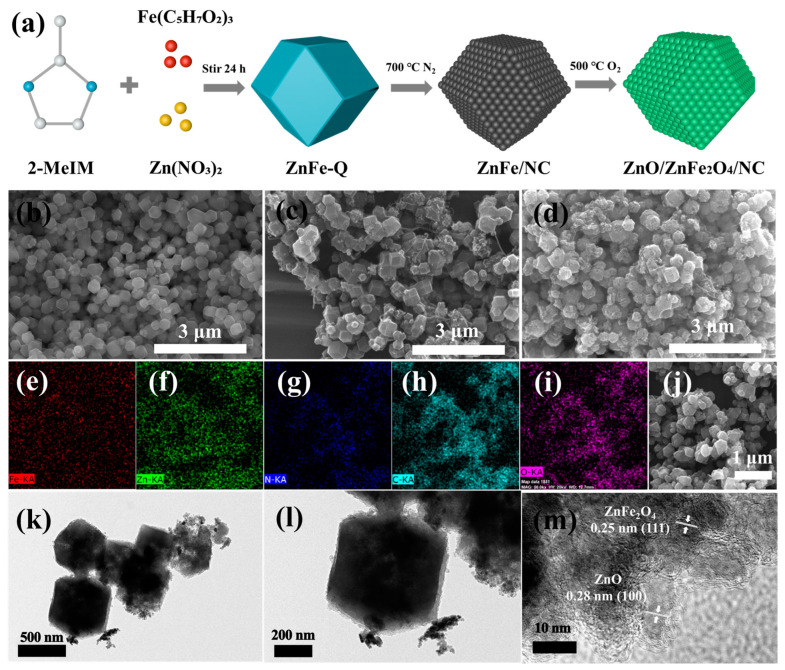
(**a**) Synthesis schematic; SEM morphology of (**b**) ZnFe complex precursor (ZnFe-Q); (**c**) ZnFe/NC; (**d**) ZnO/ZnFe_2_O_4_/NC; (**e**–**i**) EDS maps of the corresponding elements of ZnO/ZnFe_2_O_4_/NC; (**j**) original SEM morphology of ZnO/ZnFe_2_O_4_/NC corresponding to EDS; (**k**) low-resolution TEM morphology, (**l**) high-resolution TEM morphology, and (**m**) lattice fringes for ZnO/ZnFe_2_O_4_/NC.

The SEM morphology of the ZnFe complex precursor (ZnFe-Q) synthesized by precipitation is shown in Figure 1b. The precursor exhibits a uniform dodecahedral structure. Figure 1c,d show the SEM images of ZnFe/NC and the final pyrolysis product ZnO/ZnFe_2_O_4_/NC, respectively. The ZnFe/NC appears more angular than the regular dodecahedron precursor, and some carbon nanotubes are visible, indicating successful carbon enrichment. Despite undergoing two successive pyrolysis steps, the final product retained the basic dodecahedral morphology, albeit with partial adherence and melting. Energy Dispersive Spectroscopy (EDS) analysis of the ultimate pyrolysis product, ZnO/ZnFe_2_O_4_/NC, is presented in Figure 1e–i, revealing a uniform distribution of elements (Fe, Zn, N, C, and O) without agglomeration.

Furthermore, Figure 1k,l display transmission electron microscopy (TEM) images that highlight the regular dodecahedral morphology of ZnO/ZnFe_2_O_4_/NC. The dodecahedral shape is observed to be well-preserved even after the two-step pyrolysis processes, indicating the structural stability of the material. These TEM images provide valuable insights into the microstructure of ZnO/ZnFe_2_O_4_/NC, confirming the successful synthesis and retention of the desired morphology. The high-resolution TEM image in Figure 1m displays lattice fringes corresponding to the ZnO (100) and ZnFe_2_O_4_ (111) crystal planes, confirming the presence of these phases in the final pyrolyzed samples. Moreover, amorphous carbon structures can also be observed in the images. The abundant carbon structures on the surface are able to form a conductive network, enhancing the charge and Li^+^ diffusion efficiency.

**Figure 2:**
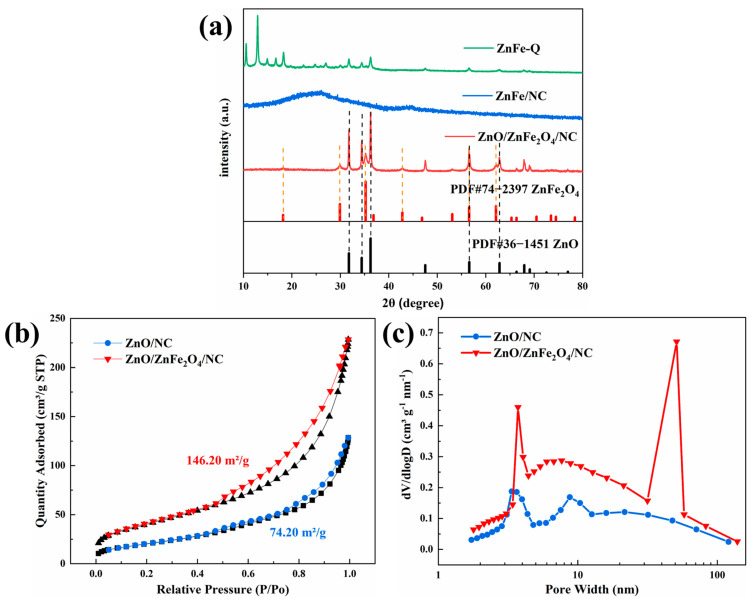
(**a**) XRD physical phase of the synthesized sample; (**b**) nitrogen adsorption and desorption isotherms; (**c**) distribution of pore size structure.

As Figure 2a shows, the X-ray diffraction (XRD) analysis correctly identified the physical phases of the synthesized samples in the scanning range of 10° to 80°. The XRD pattern of the ZnFe complex precursor (ZnFe-Q) closely matches that of the ZIF-8 host material, indicating that the addition of Fe did not alter the crystal structure. In contrast, the nitrogen-only pyrolyzed product, ZnFe/NC, primarily exhibits carbon peaks in the XRD pattern, consistent with the carbon structure observed in the SEM and TEM images. This demonstrates that pyrolysis processes can fully retain the carbon structure. The final pyrolyzed product, ZnO/ZnFe_2_O_4_/NC, is identified as a carbon and nitrogen composite of ZnO and ZnFe_2_O_4_ by comparison with standard reference patterns. The synergistic effect of the two phases plays a positive role in the subsequent electrochemical performance.

Nitrogen adsorption–desorption isotherms were used to measure the specific surface area of the synthesized samples, as shown in Figure 2b and Figure S1a. The pattern corresponds to type IV isotherms, which are characteristic of mesoporous materials. The specific surface areas of ZnO/NC and ZnO/ZnFe_2_O_4_/NC samples were measured to be 74.20 m^2^/g and 146.20 m^2^/g, respectively. This demonstrates that the mixed spinel structure formed by Fe and Zn doping of the original ZIF-8 host phase after two consecutive pyrolysis steps in different atmospheres has a positive effect on increasing the specific surface area [35,36]. The higher specific surface area provides more active sites, which lays the foundation for the subsequent enhanced electrochemical performance of ZnO/ZnFe_2_O_4_/NC. The pore size distributions of the ZnO/NC and ZnO/ZnFe_2_O_4_/NC samples are shown in Figure 2c. (For the pore size distributions of ZnFe/NC, see Supporting Information Figure S1b.) The ZnO/NC sample is predominantly mesoporous with pore sizes less than 10 nm, while the ZnO/ZnFe_2_O_4_/NC sample exhibits a bimodal pore distribution concentrated at 4 nm and 50 nm. Previous studies have shown that excellent energy storage performance of carbon matrix composites is mainly attributable to increased specific surface area from carbon groups, ensuring adequate contact between electrodes and electrolytes. Additionally, suitable pore size distributions are crucial, as micropores accelerate ion exchange while mesopores and macropores act as reservoirs [37].

**Figure 3:**
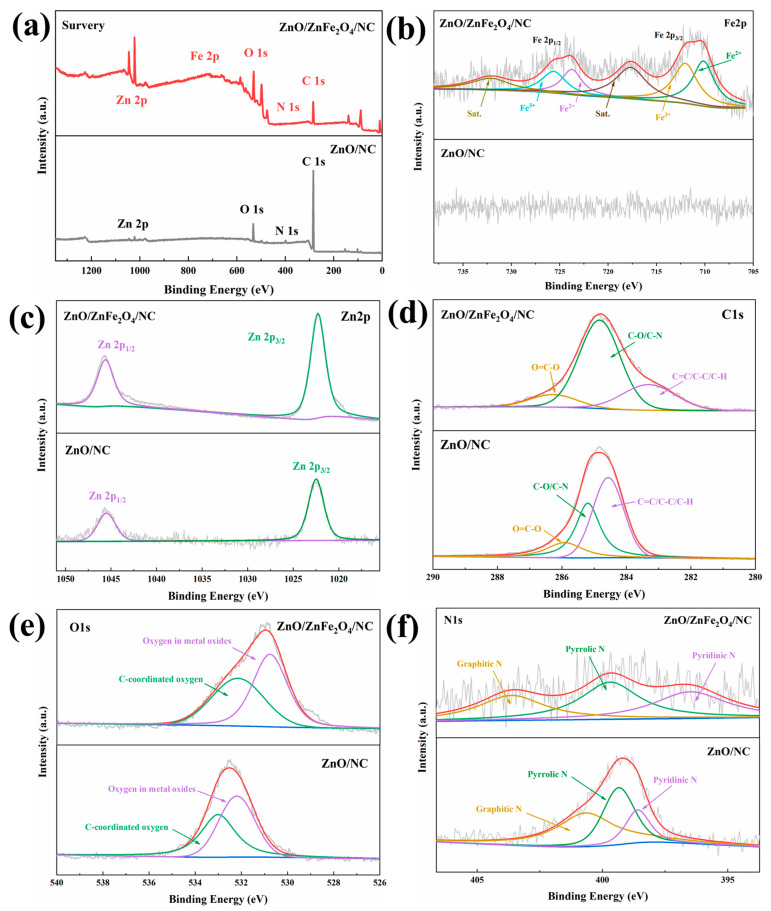
XPS spectra of ZnO/NC and ZnO/ZnFe_2_O_4_/NC: (**a**) total spectrum; high-resolution XPS spectra of (**b**) Fe 2p; (**c**) Zn 2p; (**d**) C 1s; (**e**) O 1s; (**f**) N 1s.

X-ray photoelectron spectroscopy (XPS) was employed to analyze the elemental composition of the ZnO/NC, ZnFe/NC and ZnO/ZnFe_2_O_4_/NC samples. Figure 3a and Figure S2a present the overall survey spectra with five elements detected: Fe, Zn, C, N and O. At the same time, in order to qualitatively analyze the content of metals in the samples, we conducted ICP-OES, and the obtained contents are shown in Table 1. To further examine the elemental presentations, high-resolution spectra for individual elements were obtained and fitted with split peaks (Figure 3b–f and Figure S2b–e). Figure 3b shows that in the ZnO/ZnFe_2_O_4_/NC sample, Fe is mainly divided into Fe 2p_1/2_ and Fe 2p_3/2_ peaks accompanied by Fe^3+^and Fe^2+^ valence fluctuations and two satellite peaks. In contrast, ZnO/NC does not exhibit any Fe signal. The Zn 2p_1/2_ and Zn 2p_3/2_ peaks of the Zn element in Figure 3c are positioned at 1044.63 eV and 1021.73 eV, respectively. The C 1s in both samples is characterized by three peaks representing O=C-O, C-O/C-N and C=C/C-C/C-H (Figure 3d). The corresponding peak positions for these three carbon peaks in ZnO/ZnFe_2_O_4_/NC are found at 286.28 eV, 284.83 eV and 283.33 eV, respectively, confirming the carbon-based nature of the composite. The O 1s in Figure 3e of ZnO/ZnFe_2_O_4_/NC exhibits peaks corresponding to O in metal oxides (530.08 eV) and C-coordinated O (531.53 eV). Figure 3f shows the splitting peaks of the N 1s in ZnO/ZnFe_2_O_4_/NC, which are attributed to graphite N (403.63 eV), pyrrole N (399.73 eV) and pyridine N (396.48 eV). The percentages of these various nitrogen species were calculated based on peak fitting, and the results are presented in Table 2. Numerous studies have demonstrated that the electrochemical characteristics of carbon materials are significantly influenced by the type of nitrogen species present. Graphite N is typically more brittle than pyridine- and pyrrole-based N during lithiation processes [38,39]. Therefore, the superior performance of the ZnO/ZnFe_2_O_4_/NC materials, which possess a heterojunction structure with hybridized metal oxides, can be more comprehensively explained through these findings.

### 2.2. Characterization of Electrochemical Properties

**Figure 4:**
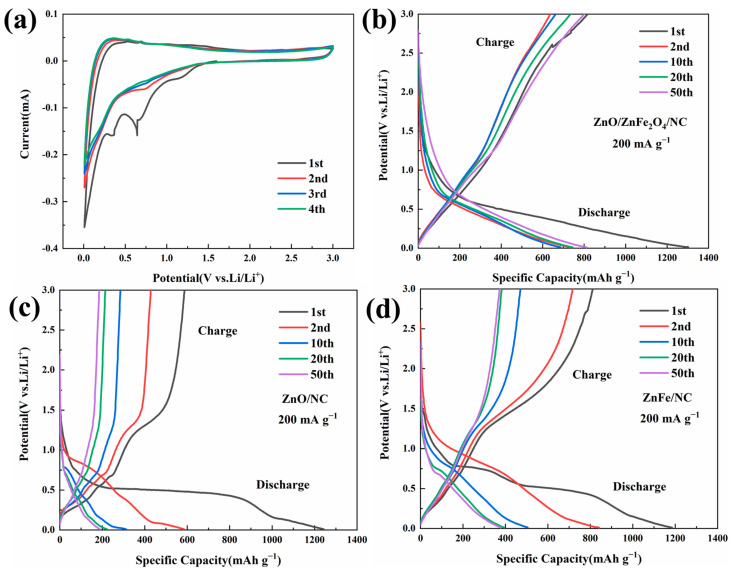
(**a**) CV curves of ZnO/ZnFe_2_O_4_/NC at 0.2 mV s^−1^ sweep rate; constant-current charge–discharge curves of (**b**) ZnO/ZnFe_2_O_4_/NC; (**c**) ZnO/NC; (**d**) ZnFe/NC.

Cyclic voltammetry (CV) curves in Figure 4a show the electrochemical reactions of the ZnO/ZnFe_2_O_4_/NC sample. After the first two cycles, the overlapping CV curves demonstrate strong electrochemical reversibility and capacity retention. During the initial cathodic scan, the first small bun peak (1.15 V) can be attributed to the embedding of Li^+^ in ZnFe_2_O_4_. Two distinct peaks can then be observed near 0.64 V and 0.35 V, which can be attributed to the decomposition of ZnO and ZnFe_2_O_4_ to form Zn^0^ and Fe^0^. The peak at about 0.01 V is usually attributed to the alloying reaction of Zn^0^ and Li^0^ and the charge accumulation [12]. In addition, we also provided supplementary explanations for CV through dQ/dV curves of the ZnO/ZnFe_2_O_4_/NC sample, as shown in the supporting information, Figure S3. Galvanostatic charge–discharge curves in Figure 4b–d compare the three samples at 200 mA g^−1^. ZnO/ZnFe_2_O_4_/NC shows a first-cycle coulombic efficiency of 62.7% and discharge capacity of 1301.8 mAh g^−1^. ZnO/NC and ZnFe/NC have lower first-cycle efficiencies of 47.4% and 68.8%, respectively, and discharge capacities of 1237.8 and 1179.4 mAh g^−1^. The low first-cycle efficiencies are attributable to irreversible SEI formation, evidenced by the discharge plateaus around 0.5 V [40,41]. However, ZnO/ZnFe_2_O_4_/NC shows superior overlap of charge–discharge curves compared to ZnO/NC and ZnFe/NC, indicating higher electrochemical stability and lower polarization.

**Figure 5:**
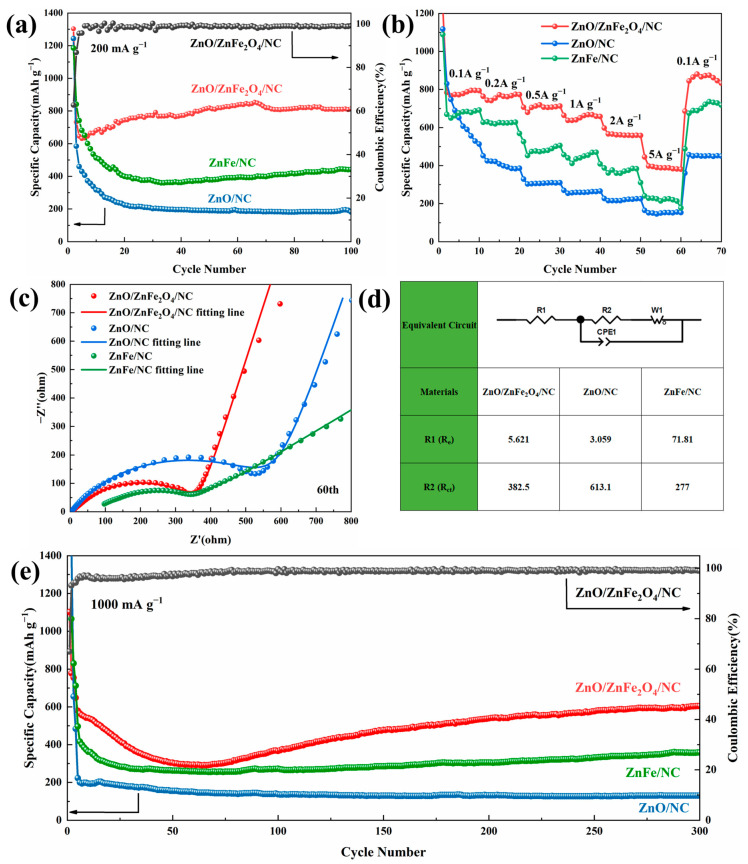
(**a**) Cycling performance curves at 200 mA g^−1^; (**b**) multiplicity performance; (**c**) Nyquist plots and fitting line of samples after 60 charge/discharge cycles at the current density of 200 mA g^−1^; (**d**) equivalent circuit and charge transfer resistance values; and (**e**) cycling performance curves at 1000 mA g^−1^.

Cycling performance is a crucial indicator of battery performance. Figure 5a presents the cycling performance graphs of various synthesized samples at a current density of 200 mA g^−1^. After 100 cycles, the specific capacity of ZnO/ZnFe_2_O_4_/NC remains stable at 812.8 mAh g^−1^, and the coulombic efficiency is consistently close to 100%. In contrast, the specific capacities of ZnFe/NC and ZnO/NC after 100 cycles are 441.2 mAh g^−1^ and 192.2 mAh g^−1^, respectively. This indicates that ZnO/ZnFe_2_O_4_/NC exhibits superior cycling stability compared to the other samples. To further evaluate the cycling stability, a long-term cycling test at a high current density of 1000 mA g^−1^ was conducted (Figure 5e). After 300 cycles, ZnO/ZnFe_2_O_4_/NC still maintains a specific capacity of 604.7 mAh g^−1^, while the specific capacities of ZnFe/NC and ZnO/NC are only 358.7 mAh g^−1^ and 130.3 mAh g^−1^, respectively. Under conditions of both low and high current densities, the ZnO/ZnFe_2_O_4_/NC maintains a leading position. During the initial stage, the capacity drop can be attributed to the formation of the solid electrolyte interface (SEI) film on the electrode surface. As the reaction progresses, a polymer-like gel layer forms on the electrode surface, capable of storing additional Li^+^ [42]. This leads to a gradual increase in specific capacity, eventually stabilizing.

The rate performance shown in Figure 5b is another critical metric for evaluating anode materials. The ZnO/ZnFe_2_O_4_/NC delivers high specific capacities of 787.8, 764.0, 706.8, 662.8, 559.3 and 387.9 mAh g^−1^ at increasing current densities of 0.1, 0.2, 0.5, 1, 2 and 5 A g^−1^, respectively. Remarkably, after the high-rate cycling at 5 A g^−1^, returning to 0.1 A g^−1^ results in an average specific capacity of 874.6 mAh g^−1^ for ZnO/ZnFe_2_O_4_/NC, even exceeding its initial 0.1 A g^−1^ capacity. This demonstrates excellent rate performance and reversibility for ZnO/ZnFe_2_O_4_/NC. In comparison, the specific capacities of ZnFe/NC and ZnO/NC at different current densities are much inferior to ZnO/ZnFe_2_O_4_/NC.

The electrochemical kinetic behavior of the battery was evaluated using electrochemical impedance spectroscopy (EIS) technology. Figure 5c shows the Nyquist plot derived from the EIS of the ZnO/ZnFe_2_O_4_/NC, ZnO/NC and ZnFe/NC samples after 60 charge/discharge cycles at a current density of 200 mA g^−1^. They were fit to an equivalent circuit (see Figure 5d), where R1 is the electrolyte resistance R_e_, R2 is the charge transfer resistance R_ct_, CPE1 is a constant phase element and W1 is assigned to the infinite Warburg diffusion impedance. The fitting parameters obtained are shown in the table. After 60 charging/discharging cycles, the R_ct_ value (382.5 Ω) of ZnO/ZnFe_2_O_4_/NC is lower than that of ZnO/NC (613.1 Ω), indicating that the charge transfer rate of ZnO/ZnFe_2_O_4_/NC is higher than that of ZnO/NC. In addition, although the R_ct_ value (277 Ω) of ZnFe/NC is smaller than that of ZnO/ZnFe_2_O_4_/NC, its electrolyte resistance R_e_ is 71.81 Ω, much higher than that of ZnO/ZnFe_2_O_4_/NC (5.621 Ω).

By comparing the cycling and rate performance of ZnO/ZnFe_2_O_4_/NC to other samples, it is evident that ZnO/ZnFe_2_O_4_/NC delivers superior specific capacity at various current densities. This can be attributed to the higher specific surface area of ZnO/ZnFe_2_O_4_/NC providing more lithium storage sites. The excellent cycling stability of ZnO/ZnFe_2_O_4_/NC also stems from its better structural durability, as the synergistic effects of the ZnO and ZnFe_2_O_4_ double oxides can better buffer volume changes during cycling, preventing structural damage. Furthermore, the heterostructure enhances the energy storage performance through improved structural stability and electronic conductivity enabled by charge redistribution [43,44]. Overall, the higher specific surface area, improved structural stability and charge redistribution in the heterostructure collectively contribute to the superior specific capacity, cycling stability and rate performance of ZnO/ZnFe_2_O_4_/NC compared to other samples.

## 3. Experimental Section

### 3.1. Material Preparation

The raw materials used in the experiment were purchased from Aladdin Chemical Reagents Ltd. and were of analytical grade, ready for immediate use.

Figure 1a depicts the schematic diagram of sample preparation. The precursor Zn/Fe hybridization product (ZnFe-Q) was first synthesized via a facile precipitation method. Specifically, 2.38 g Zn(NO_3_)_2_ was dissolved in 80 mL of methanol, while 5.26 g of 2-methylimidazole and 5.64 g of ferric acetylacetonate were dissolved in 160 mL of methanol. The atomic ratio of Zn and Fe is 0.8497. The Zn(NO_3_)_2_ solution was rapidly poured into the 2-methylimidazole and ferric acetylacetonate solution, producing ZnFe-Q after constant stirring for 24 h and centrifugal washing. The product was vacuum dried overnight.

The dried product ZnFe-Q was then carefully ground and calcined at 700 °C for 2 h with a heating rate of 2 °C min^−1^ in N_2_ atmosphere. Afterward, the crucible was allowed to cool naturally. This yielded a black powder, ZnFe/NC, which was collected after grinding.

Finally, the ZnFe/NC powder was flattened in a crucible and calcined in air at 500 °C for 2 h to obtain the final product, ZnO/ZnFe_2_O_4_/NC. For comparison, ZnO/NC was prepared identically but without adding ferric acetylacetonate during precursor synthesis.

### 3.2. Material Characterization

A Rigaku MiniFlex 600 X-ray powder diffractometer (Tokyo, Japan) was used to examine the physical phase. A scanning electron microscope (MLA650, Washington, DC, USA) and a transmission electron microscope (JEM-2100F, Tokyo, Japan) were used to examine the morphology and structure of the samples. The X-ray photoelectron spectrometer used was the ESCALAB 250 (London, UK) from Shanghai Uzonglab (Shanghai, China). Micrometrics ASAP2020 (New York, NY, USA), a specific surface area tester from McMurray Tick, was used to measure the specific surface area and pore size. The content of metal elements was determined by inductively coupled plasma optical emission spectroscopy (ICP-OES), Optima 5300 DV, Upton, MA, USA.

### 3.3. Electrochemical Testing

The working electrode was prepared by mixing 70% of active material, 20% of conductive agent acetylene carbon black (super P) and 10% of polyvinylidene fluoride (PVDF), which were dissolved in *N*-methyl-2-pyrrolidone (NMP) solvent to form a slurry. The slurry was coated onto copper foil to prepare the anode electrode and dried in a vacuum oven at 120 °C overnight. The loading capacity of the active substance is 0.84 mg. Lithium metal foil served as the counter electrode, 1 M LiPF_6_ in 1:1 of dimethyl carbonate (DMC): ethylene carbonate (EC) was used as the electrolyte, and a Celgard 2400 microporous polypropylene membrane was used as the separator. After assembling CR2016-type button cells in an Ar-filled glovebox, the cells were allowed to rest for 12 h before electrochemical testing. The constant current charge–discharge cycling and rate performance were evaluated between 0.05 and 3.0 V using a LAND battery tester (from Wuhan Jinnuo Electronics Co., Ltd. Wuhan, China). Cyclic voltammetry (CV) and electrochemical impedance spectroscopy (EIS) were conducted using a Metrohm Autolab PGSTAT 302N (Herisau, Switzerland) electrochemical workstation. The scanning rate of the cyclic voltammetry (CV) curve is 0.2 mV s^−1^, and the voltage range is 0.01–3.0 V. Electrochemical impedance spectroscopy (EIS) testing was carried out, with a frequency range of 0.01 to 10^5^ Hz.

## 4. Conclusions

In summary, the bimetallic oxide carbon and nitrogen composites ZnO/ZnFe_2_O_4_/NC were successfully prepared using the ZIF-8 MOF as a precursor. The solution reaction with added Fe, followed by two-stage pyrolysis, resulted in the bimetallic transition metal oxide heterojunction structure of ZnO and ZnFe_2_O_4_. This nanocomposite not only maintains the high specific surface area and morphological stability of the parent MOF, but also exhibits porous metal/carbon composite features. Given the synergistic effects of the ZnO and ZnFe_2_O_4_ components, ZnO/ZnFe_2_O_4_/NC demonstrates excellent electrochemical performance. It delivers a high discharge capacity of 1301.8 mAh g^−1^ at 200 mA g^−1^ at and retains a reversible capacity of 604.7 mAh g^−1^ after 300 cycles at 1000 mA g^−1^. The rate performance is also superior across various current densities. The ZnO/ZnFe_2_O_4_/NC nanocomposite shows great promise as a high-performance anode material. In addition, the facile synthesis method enables the creation of unique heterogeneous nanostructures with micro- and nano-scale morphologies for diverse nanomaterial applications beyond energy storage.

## Figures and Tables

**Table 1:** Content of metals in samples (%).

	Fe	Zn
ZnO/NC	-	71.24
ZnFe/NC	1.73	30.68
ZnO/ZnFe_2_O_4_/NC	1.69	29.66

**Table 2:** Percentage of N content in different forms (%).

	Graphite N	Pyrrole N	Pyridine N
ZnO/NC	48.2	34.9	16.9
ZnFe/NC	17.72	46.61	35.66
ZnO/ZnFe_2_O_4_/NC	28.8	38.3	32.9

## Data Availability

Data are contained within the article and Supplementary Materials.

## Supplementary Materials

The following supporting information can be downloaded at: https://www.mdpi.com/article/10.3390/molecules28227665/s1.
