# Supplementary material for: Synthesis and Characterization of Zinc/Iron Composite Oxide Heterojunction Porous Anode Materials for High-Performance Lithium-Ion Batteries
Source: Molecules. 2023 Nov 19;28(22):7665. doi: 10.3390/molecules28227665 (PMC10674232; doi:10.3390/molecules28227665)
Supplement: Supplementary file 1 [file molecules-28-07665-s001.zip › molecules-2658407-supplementary.pdf]

Supporting information

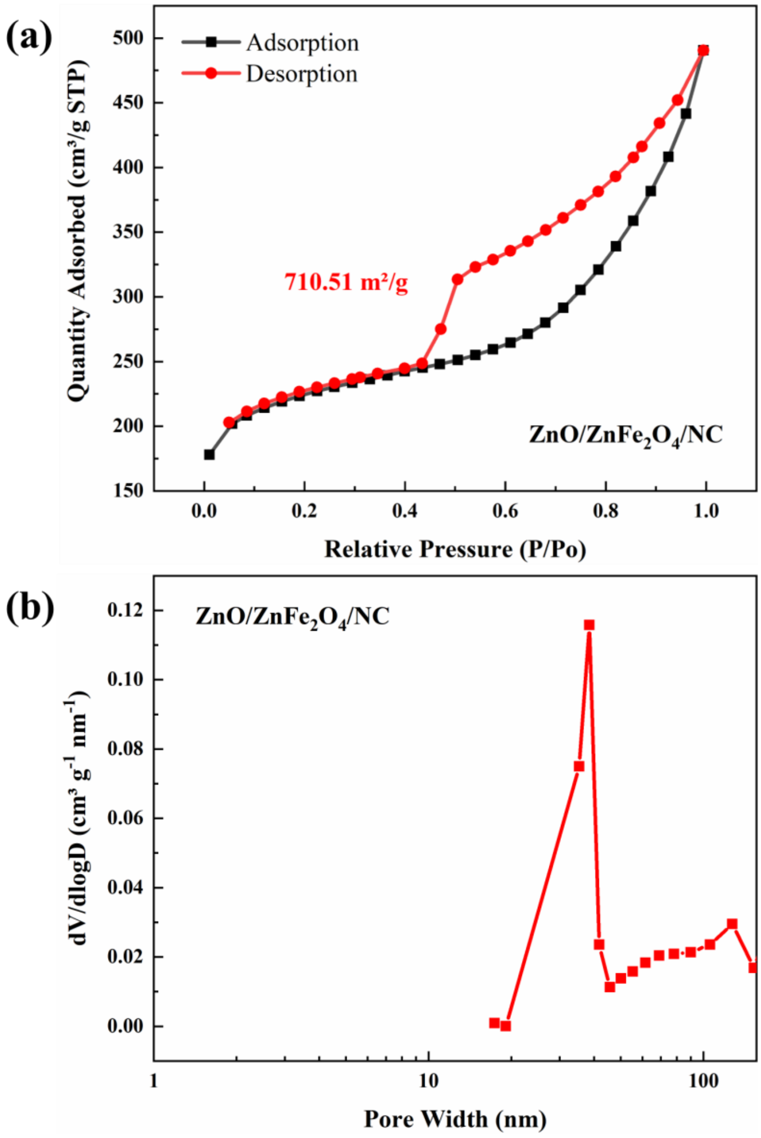

Figure S1. (a) Nitrogen adsorption and desorption isotherms; **(b)** distribution of pore size structure of ZnFe/NC

The specific surface area of ZnFe/NC is 710.51 m^2/g, and the pore size distribution is mainly composed of mesopores at 38.45 nm and macropores at 127.55 nm. The reason why the specific surface area of $ZnO/ZnFe_2O_4/NC$ is smaller than that of ZnFe/NC may be due to the gradual occupation of large pores during the crystallization process of metal oxides. However, the reason why the electrochemical performance of $ZnO/ZnFe_2O_4/NC$ is superior to that of ZnFe/NC is that it increases the synergistic effect of ZnO and $ZnO/ZnFe_2O_4/NC$ bimetallic oxides on the basis of ZnFe/NC carbon nitrogen materials.

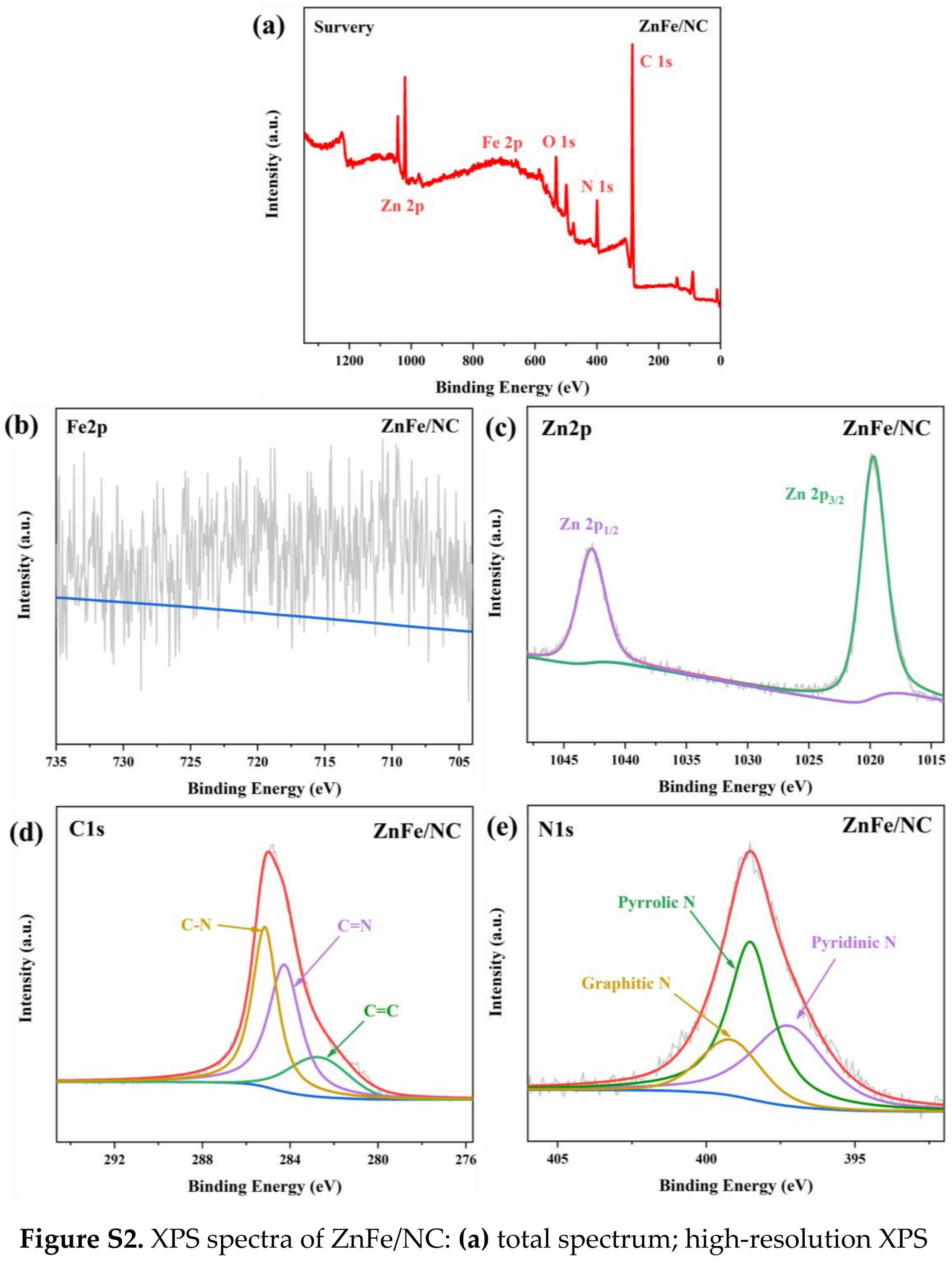

Figure S2. XPS spectra of ZnFe/NC: **(a)** total spectrum; high-resolution XPS spectra of **(b)** Fe 2p; **(c)** Zn 2p; **(d)** C 1s; **(e)** N 1s.

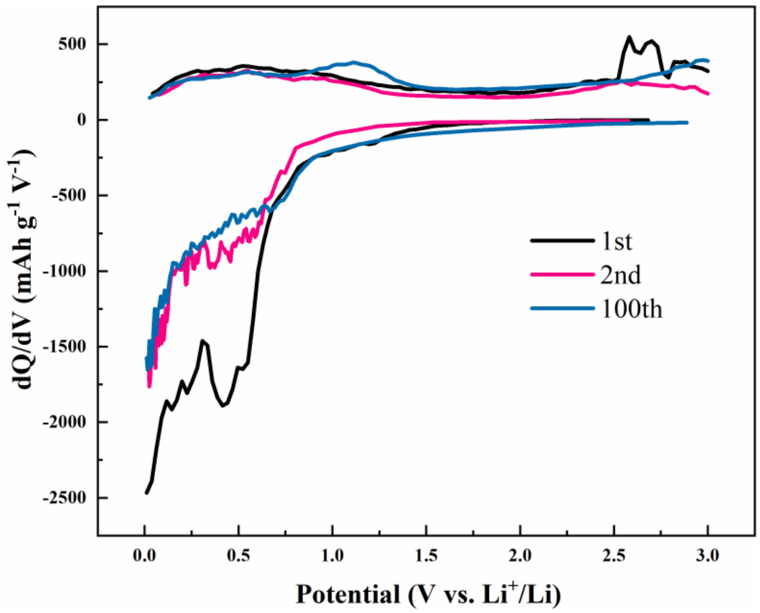

Figure S3. dQ/dV curve of $ZnO/ZnFe_2O_4/NC$

The differential capacity diagram (dQ/dV) is shown in Figure S3. For $ZnO/ZnFe_2O_4/NC$ samples, it is evident that the trend is similar to the CV cycle. Except for the first cycle, the subsequent cycles basically overlap, indicating that a Li^+ storage reaction occurred in the first cycle. During the first cycle of $ZnO/ZnFe_2O_4/NC$ (Figure 4a in the main text), the cathodic peaks at approximately 0.64 and 0.35V can be attributed to the decomposition of ZnO and $ZnFe_2O_4$ to form Zn^0 and Fe^0.
